# Transactions

**Published:** 1889-09

**Authors:** 


### Transactions.

We have just received the transactions of the twenty-fifth annual meeting of the Illinois State Dental Society.

The amount of work done at that meeting will be proximately comprehended when it is stated that the matter fills one hundred and sixty-four pages of closely printed matter.

This is one of the most active and effective state dental societies in the country, At its annual meetings a large amount of good work is done. At this meeting quite a number of good papers were read and discussed, and well discussed.

The work accomplished by such an organization is incalculable. The good accruing to mankind from the earnest and well directed effort of such a body of scientific men as composed this society for twenty-five years, is incalculable. Long may this Society live and prosper in its work.

